# Employing a Coaching Model of Supervision During Physiotherapy Placements: A Qualitative Study of the Practice Educator Experience

**DOI:** 10.1002/pri.70275

**Published:** 2026-06-30

**Authors:** Sarah Smith, Adele Anderson, Simon Godley, Paul K. Miller

**Affiliations:** ^1^ School of Health and Life Sciences Teesside University Middlesbrough UK; ^2^ North Cumbria Integrated Care Carlisle UK; ^3^ Institute of Health University of Cumbria Carlisle UK

**Keywords:** mentoring, physical therapists, qualitative research

## Abstract

**Background and Purpose:**

The current expansion of Allied Health Profession training places in the UK presents significant challenges for clinical placement provision. Coaching and Peer‐Assisted Learning (C‐PAL) models have shown success in helping reduce placement pressure in nursing and midwifery, but there is limited evidence around how such approaches might inform traditionally one‐to‐one physiotherapy placements. Addressing a gap in C‐PAL research, which has largely focused on nursing and midwifery, and on learner rather than educator experience, this exploratory qualitative study examined the experiences of Practice Educators (PEs) in implementing a C‐PAL intervention during physiotherapy placements in one UK National Health Service trust.

**Methods:**

An exploratory qualitative study was conducted using semi‐structured interviews and Reflexive Thematic Analysis. Ten PEs (seven female, three male; mean 9 years PE experience) participated. All had experience of supervising learners in traditional placements. With institutional ethical approval, interviews were conducted online, recorded and transcribed.

**Results:**

Four themes emerged: (1) ‘Oversight and Management’—initial concerns about managing multiple learners diminished when PEs observed peer support creating effective self‐monitoring mechanisms. (2) ‘Teamworking and Learning’—a shift emerged from direct teaching to facilitating peer learning, with the collaborative environment viewed as accelerating professional development. (3) ‘Patient Contact’—increased therapeutic contact time and continuity of care were highlighted, though direct supervision of clinical skills was more challenging. (4) ‘Collaborative Working and Support Networks’—the intervention facilitated collaborative relationships, though required additional administrative effort.

**Discussion:**

Participants found that C‐PAL offered a promising alternative to traditional physiotherapy placement supervision, potentially addressing educational capacity challenges while promoting greater learner autonomy and interdisciplinary skills. They also highlighted complex challenges emerging from managing multiple learners across locations and coordinating with colleagues. Preliminary findings suggest that C‐PAL approaches may offer potential for expanding placement capacity while maintaining educational quality, though this warrants investigation across a wider range of clinical contexts. Sufficient preparation time, clear role delineation, and educator development focused on facilitation skills are likely to be important enabling conditions.

## 1 Introduction

The ongoing expansion of Allied Health Profession (AHP) training places in the UK, as outlined in the 2023 National Health Service (NHS) Long Term Workforce Plan (NHS England 2023), presents significant challenges for clinical placement provision. The proposed 50% expansion in university places aims to address medium‐to‐long term service needs but raises substantial questions about whether there will be enough practice placements, and trained Practice Educators (PEs) available to support it (St. John‐Matthews and Hobbs 2020). This is particularly acute in physiotherapy where the Chartered Society of Physiotherapy (CSP) has a standing requirement for 1000 h of practice‐based learning, which has traditionally relied upon a one‐to‐one model apprenticeship‐style mentoring from PEs (The Chartered Society of Physiotherapy 2019; Twogood et al. 2020). Within this model, experienced PEs directly supervise individual learners while maintaining connections with academic institutions (The Chartered Society of Physiotherapy 2019, 2021). This approach, while historically effective for developing clinical competencies, is also highly labour‐intensive. With increasing learner numbers straining an already limited PE capacity, healthcare providers and higher education institutions (HEIs) have been tasked with developing innovative placement approaches that maintain educational quality while addressing resource constraints.

One promising alternative to traditional mentoring models is the coaching and peer‐learning approach, sometimes called C‐PAL (Wareing et al. 2018), which has shown success across various healthcare disciplines, particularly nursing and midwifery (Hill et al. 2022; Subramaniam et al. 2015; Vuckovic and Landgren 2021; Wareing et al. 2018). This approach typically involves learners working collaboratively in small groups, sharing decision‐making responsibilities while supervised by a PE who adopts a ‘coaching’ role rather than providing direct apprenticeship‐style supervision (Markowski et al. 2022b; Markowski et al. 2022a; Vuckovic et al. 2019; Vuckovic and Landgren 2021; Wareing et al. 2018). PEs in this model focus on facilitating self‐directed learning through structured feedback and reflection (Hill et al. 2022; Dyk et al. 2022), manifestly designed to ‘unlock’ learners' potential. Central to this process is working with learners to set learning goals, longitudinally manage those goals in response to evolving circumstances, and find the actions necessary to pragmatically achieve them (Wareing et al. 2018). International evidence, particularly from nursing education, suggests this approach can effectively support clinical skill development while making more efficient use of PE resources (Markowski et al. 2021; Williamson et al. 2020). This body of research has also indicated strongly positive manifest outcomes, with key latent impacts upon teamworking skills, peer‐support mechanisms and adaptations to intra‐group leadership matters (Anderson et al. 2024; Smith et al. 2025). However, the physiotherapy context presents distinct challenges that limit the straightforward applicability of findings from nursing and midwifery. The CSP's requirement for 1000 h of practice‐based learning, and the profession's longstanding reliance on a one‐to‐one supervisory model mean that evidence of C‐PAL's feasibility must be developed within this specific professional context rather than inferred from adjacent disciplines.

In response to these challenges, and with reference to the evidence above, a trial intervention was implemented in one UK NHS trust, wherein final‐year physiotherapy learners from both undergraduate and postgraduate programs worked in small groups under a single PE's supervision. This approach, adapting Wareing and colleagues' Coaching and Peer‐Assisted Learning (C‐PAL) model (Wareing et al. 2018), employed the GROW framework to structure learning experiences through daily logs and reflective practice (Smith et al. 2025; Wareing et al. 2018). A 6‐week placement period integrated peer learning with coaching supervision, representing a significant departure from traditional one‐to‐one mentoring.

As noted above, the bulk of existing C‐PAL research focuses on the learner experience, or on combined learner and PE perspectives, tending to foreground overall placement efficacy and learner satisfaction (Markowski et al. 2022a; Subramaniam et al. 2015; Vuckovic et al. 2019; Vuckovic and Landgren 2021; Wareing et al. 2018). This pattern is reflected in the companion paper emergent of the present intervention (Smith et al. 2025). Thus, at the time of writing, this paper occupies a novel position in terms of addressing an issue hitherto rarely addressed in physiotherapy, and not at all from a PE perspective. The analysis is informed by Communities of Practice theory (Wenger 1998), which provides a lens through which PE role reorientation and the progressive development of learner autonomy can be understood as processes of legitimate peripheral participation within clinical settings.

Understanding PE experiences is crucial in this respect, as their insights can inform the development and refinement of coaching and peer‐learning approaches in physiotherapy education, particularly as the profession adapts to meet growing workforce demands. Additionally, PE perspectives on managing mixed groups of undergraduate and postgraduate learners prospectively offer valuable insights for future placement design.

## 2 Methods

### 2.1 Design

A qualitative study was conducted using semi‐structured interviews. This approach is optimally suited for collecting and interpreting professional views and reflection (Cuthbertson et al. 2020), particularly where participants are asked to unpack and make sense of a new experience (Smith et al. 2025). The study was conducted in accordance with the Declaration of Helsinki and is reported following the Consolidated Criteria for Reporting Qualitative Research (COREQ) checklist.

### 2.2 Participants

A total of *N* = 10 PEs involved in the coaching trial volunteered and met the criteria for participation in the research. Of these, *n* = 7 identified as female and *n* = 3 identified as male. The mean years of experience as a PE was 9 years, with a range of 4–18 years. All PEs had previous experience of supervising learners in traditional one‐to‐one placements. Conditions of ethical approval delimit more explicit demographics regarding participants being detailed herein, given the strong potential for identification among peers involved in the study. None of the participating PEs withdrew from the research.

It should be noted that participation was voluntary; the implications of this for the representativeness of the sample are discussed in the Limitations section.

### 2.3 Data Collection

The first author (henceforth A1) and the fourth (A4) prepared a semi‐structured interview schedule that was designed to facilitate open exploration of the participants' experiences of implicating the coaching model, using open questioning and prompts to elicit accounts of specific experiences rather than general observations. This was further checked and edited by A2 and A3, and the final version is available as Supporting Information S1: Supplement 1.

All interviews were conducted and recorded online using Microsoft Teams, within 4 weeks of the supervised learners completing their placements, and participants were made aware that their interviewer (A1) was a qualified physiotherapist. Open questioning encouraged participants to address their experiences personally rather than formally. Iterative interviewing, with prior responses informing latter prompts, was employed across the course of the data collection. The mean interview duration was approximately 31 min. The media files were transcribed verbatim, and then redacted for participant identity protection by a trusted agent who signed up to the full conditions of ethical approval given. The redacted transcripts were then reviewed for robustness against the original media files by A1.

### 2.4 Data Analysis

Following the provisional work done by A1 and A4, data analysis was undertaken using the latter five of the six steps of reflexive thematic analysis outlined by Braun and Clarke (2006), (2022). The operation of these is schematised in Table 1.

**TABLE 1:** Analytic process.

Step	Actions taken
1. Generation of codes	A1 and A4, drawing upon their familiarity with the full data corpus, each developed a set of initial codes which they then reviewed against the original transcripts. Coding was conducted at a semantic level, focussing on explicitly stated content rather than latent meaning, consistent with the study's applied orientation. Where the codes developed by A1s and A4 initially diverged, disalignments were resolved through structured discussion across analytical meetings in which the two researchers clarified their codes until consensus was met.
2. Search for themes	From the agreed codes, A1 and A4 collaboratively developed a set of *n* = 17 subthemes. These were provisionally aggregated into *n* = 4 major themes that were taken to best describe the participants' experiences.
3. Reviewing themes	All major themes were reviewed on two key levels. Firstly, specific data corresponding with each theme was retroactively spot‐checked by A1 and A4 to determine the coherence of fit. At this stage, no changes to the thematic structure were deemed necessary. Secondly, each major theme was considered in relation to the full data corpus. This was a recursive process, conducted by all authors. While no significant changes to the thematic structure were deemed necessary at this point, the discussion was critical for stage [4], in terms of rendering the analysis clear for a wider physiotherapy audience.
4. Defining and naming themes	An overall narrative for the data was considered in the naming of themes by all authors, drawing upon their professional and intellectual backgrounds. In these terms, key considerations were (firstly) faithfulness to the participant voice, followed by accessibility for a broad physiotherapy readership.
5. Report writing	The final outcome was written as a conversation between all authors, with a view to maximum impact in the intended field of dissemination.

Data sufficiency was assessed iteratively: following eight interviews, no substantive new codes emerged from the data, and the analytical team collectively agreed that the conceptual terrain relevant to the research focus had been adequately mapped. The near‐total sampling of the available population provides additional grounds for confidence in this assessment. The Communities of Practice framework informed interpretation throughout, particularly in attending to how participants described shifts in role, relational positioning, and the structuring of learning environments.

Theme development was fully inductive; codes were grouped into sub‐themes on the basis of conceptual similarity, and sub‐themes were subsequently aggregated into major themes via examination of patterns of co‐occurrence and conceptual relationships across the data corpus. Supporting Information S1: Supplement 1 (the interview schedule) constitutes a partial audit trail for the analytical process. Preliminary findings were additionally presented at a key UK physiotherapy conference, where critical engagement from peers contributed to the refinement of the final thematic structure. Particular analytic attention was paid to instances that sat in tension with the dominant patterns within each theme; rather than being minimised, these were retained and treated as cases qualifying the limits of the theme's applicability, and are reported as such below.

### 2.5 Research Team and Reflexivity

This investigation was conducted by a research team comprising physiotherapy practitioners and academics. Two of the researchers, A1 (a female chartered physiotherapist and academic) and A2 (a chartered female physiotherapist) were directly involved in the trial's implementation. A3 (a chartered male physiotherapist and academic) and A4 (a male social psychologist with extensive expertise in qualitative health research) provided external perspectives on the design and/or analysis. In terms of relationships with participants, A1 and A3 were academic tutors at the lead HEI during the research, and A2 was the clinical lead in the partner trust. All therefore had prior professional relationships with the participants, while A4 did not.

The prior professional relationships held by A1, A2, and A3 with participating PEs constituted a methodological consideration insofar as participants may have moderated their accounts in response to perceived professional or institutional expectations. The use of open questioning was intended in part to mitigate this risk. Nevertheless, given that interviews were conducted by A1, it is acknowledged that the potential for social desirability biases (Bergen and Labonté 2020) cannot be fully eliminated in this context, and this should be borne in mind when interpreting the findings. Reflexive practice was maintained throughout analytical team discussions at which interpretive assumptions were surfaced and interrogated, and through A4's external positioning, which provided a check on readings that might otherwise have been shaped by insider familiarity with the clinical context. In more conventional terms, credibility was addressed through iterative interviewing and independent coding by two researchers.

Consonant with the key conditions for evaluating trustworthiness in qualitative research outlined by Yardley (2000), the ‘transparency and coherence’ of the analysis were handled by ensuring that all emerging concepts were represented alongside clear qualitative evidence in support of their veracity. To help safeguard the ‘impact and importance’ of the work, the findings were taken to a key UK physiotherapy conference. Feedback attained from this conference helped formulate the final analysis and discussion. Dependability was managed through the maintenance of an analytical record across the research process, and confirmability through the systematic anchoring of all analytic claims to direct participant data, as evidenced in the Results section below.

### 2.6 Ethical Considerations

This study received full ethical approval from the Research Ethics Panel at A1's institution, reference 20/04. All data were handled strictly in line with the conditions given.

## 3 Results

Thematic analysis of data yielded four global themes:

1. Oversight and Management
2. Teamworking and Learning
3. Patient Contact
4. Collaborative Working and Support Networks

These themes are elucidated below with reference to direct data where it is illustrative. Quotes are presented verbatim and not ‘corrected’ for grammar to retain a sense of the original speakers' voices to the highest possible degree. Where an edit or abridging is made for sense or relevance, square brackets or ‘…’ are used.

### 3.1 Oversight and Management

The initial supervisory anxieties expressed by PEs illuminate the extent to which the one‐to‐one model had become the unspoken baseline against which any departure was measured. What the data below reveal, however, is that these anxieties were largely modified through experience rather than validated by it:

P4: Initially, there was some apprehension ‘cos it was difficult to visualise how it would work… it’s particularly heavy on the MDT side.

P6: I was extremely anxious… How would I keep an eye on four people when usually it’s difficult to keep an eye on one person and how would I manage to give good feedback for four students?

P8: I think as a Team Lead that’s only natural. And I mean the reservations were very much around competence, quality.

However, these apprehensions diminished as educators observed how peer support created effective self‐monitoring mechanisms:

P6: I actually find that my workload is less, because I don’t have to do the clinical reasoning as much out loud quite as often with my students as I find they’re doing it amongst themselves.

P7: I think it works really well. When you have a good group of students that work nicely together, and I think rotating them so they’re working with different people… really works.

While local experiences of the coaching intervention where overwhelmingly positive, logistical difficulties emerged around managing multiple learners across different physical spaces and coordinating with other healthcare professionals:

P3: Logistically it’s maybe a little more challenging ‘cos of the number of therapists around and kind of ensuring that…one of the things I’m currently learning is ensuring they kind of pair up together rather than getting split off.

This logistical strain was not uniform across contexts. For P3, interprofessional configurations disrupted the self‐monitoring dynamic that otherwise eased oversight by marking a boundary condition on the theme rather than an arbitrary footnote to it:

Particularly…the working with the occupational therapists…it’s a little bit more organisational skills involved in because there’s two of them erm…with a TI working so there’s a lot more sort of delegating skills that need to be.

### 3.2 Teamworking and Learning

The data below suggest not only a logistical adjustment among participants, but a substantive reorientation in how they understood their professional role. Central to this was a move from the direct transmission of clinical knowledge to the organisation of conditions in which peer‐mediated learning could take place:

P2: We talked a lot about kind of how we were going to do that within the team…and that’s probably the best thing that’s come out of it really, teamworking.

P6: When you’ve got four, they are constantly bouncing ideas off each other and I just have to listen most of the time.

P7: You can send two of them in to see a patient and you can kind of just earwig from the side of the room and kind of, if you know the level of your students, you can kind of send them to appropriate patients.

This collaborative environment was also viewed by participants to have accelerated professional development among their charges:

P2: It gives them the opportunity to take on team working and take more ownership of their own learning.

P6: As a result, they’re much more confident, they have got the skills that you’d expect a band five to have, they’re happy to take the lead…and the students with less experience are questioning the more senior students, they’re [all] getting lots from it.

One account, however, complicates the otherwise consistent picture of frictionless peer learning. Where it might be reasonable to assume a shared trajectory toward full participation, P1's reflection points to the work required to hold learners at differing levels within a single community of practice:

P1: I know, in the first few weeks it was, not challenging but I think ‘cos we had students that were at different levels I think there was, not, it wasn’t overly challenging to overcome but it was just something that we had to be aware of.

### 3.3 Patient Contact

The increased therapeutic contact time for learners, and the benefits thereof, were consistently highlighted across the participants' interviews. The data below do not only document increased contact time as an outcome of the model; they reveal a reconfiguration of the learner‐patient relationship. Ultimately, sustained engagement was routinely seen to replace episodic supervision as the organising principle of clinical learning.

P3: I think from our service point of view…it’s helped us [because they’ve] been …seeing the bulk of the caseload.

P6: The patients are getting longer rehab and better rehab.

P8: There’s certainly one that jumps to mind that has been able to be seen two or three times a week, because there’s the two of [the students].

Continuity of care was also notably seen to have improved under the coaching model:

P1: Because they’ve known the plan, and they’ve known about them… they’ve definitely benefitted from probably a clinical point of view because both students have known where they’re going…They’ve had that full patient journey cos they’ve been seeing patients from day one.

This expansion of contact, however, generated a tension specific to the physiotherapy context. The same configuration that multiplied therapeutic contact and continuity simultaneously withdrew the PE from the moment‐by‐moment visual supervision on which hands‐on skills assessment depends. This was a trade‐off of little consequence in disciplines without an equivalent manual‐handling requirement but directly consequential here:

P7: When you’re working together quite often, I would then go and see a different patient. And if I’m not directly supervising them or if they’re behind curtains, I can’t do that real time updating of their manual handling skills unless I sneak round the corner into the curtains, which can be a bit off‐putting [for them].

### 3.4 Collaborative Working and Support Networks

Themes 2 and 4 are distinguished here by direction rather than degree: where Theme 2 concerned learning generated within the learner group, the present theme concerns the external relationships (PE, university, MDT, service structures) that determined whether that internal dynamic could be sustained. The interplay between PEs, university staff, MDT colleagues and service development structures was seen to determine whether the model could be sustained beyond the immediate supervisory relationship:

P6: They’re happy to go into handover, they’re happy to do delegation, they’re happy to supervise their other students.

P2: It meant they got to spend time with different members of the MDT… that feedback got given back to our physio team.

The educational partnership between clinical educators and university staff also proved crucial for implementation:

P4: The university has definitely always been quite responsive… they’re always happy to come along to the group supervisions…with being part of the first pilot… we kind of got a lot of support from the uni…we got a bit of training before we got the students.

Service development projects further emerged as a key active and potential collaborative activity:

P5: In our area we’ve been developing a Service Development Project in which all the students so far have taken a role and developed that sort of lasting legacy on our ward.

P1: We’ve kind of been situated on…one side of Critical Care mainly and I think that’s been really good for the students. It’s led their continuative care.

However, coordinating these networks was noted to require additional administrative effort:

P4: I think in some respects it can be more time consuming, in like the kind of the admin side of it…the preparing for them all to come, which I think I’m getting slicker at.

## 4 Discussion

This study presents a novel qualitative analysis of PEs' experiences of implementing a coaching model of supervision during physiotherapy placements. The findings illuminate several matters of both practical and theoretical importance for the future development of placement education in physiotherapy and, potentially, allied healthcare more broadly.

The observations regarding oversight and management draw attention to how PEs' initial anxieties about supervising multiple learners—particularly around patient safety and quality of supervision—were largely dispelled through practical experience. This finding aligns with Dennis and colleagues' work on simulation‐based placements in Australia (2022), in which learners also reported high satisfaction and competency outcomes with teams of eight peer‐supported learners to one supervisor. While some concerns around the logistics of managing learners across different spaces remained pertinent throughout, the peer‐support mechanisms that emerged *between* learners reduced many of the anticipated supervisory pressures. Although a novel finding in extant physiotherapy research, this is supported (to a limited extent) by evidence in nursing, which similarly (albeit tentatively) indicates that placement designs based upon coaching and/or peer‐supported learning—by a variety of labels—can ultimately lighten the supervisory load for PEs (Clarke et al. 2018; Williamson et al. 2020).

The shift from direct teaching to learning facilitation evidenced in this study reflects a broader transformation in how PEs understood their educational role. Rather than simply ‘delivering’ knowledge in a classical apprenticeship model, PEs found themselves coordinating more sophisticated learning environments in which learners could develop clinical reasoning skills through peer interaction (Hill et al. 2022; Markowski et al. 2022b; Smith et al. 2025; Tweedie et al. 2019). This appears to have accelerated professional development in ways that traditional one‐to‐one supervision might not. As Wareing and colleagues have noted in their study of nursing education (2018), this transformation in the supervisory approach can lead to enhanced learner autonomy and clinical confidence. Future training for physiotherapy PEs around delivery of this order of placement would likely benefit from a focus on this order of facilitation skills.

Perhaps most strikingly in the physiotherapy domain, the coaching model was not only seen by participants to have facilitated more overall direct interaction between learners and patients, which it was manifestly designed to do, but also to have improved continuity of care through sustained learner engagement with individual cases. Markowski and colleagues have reported similar benefits in midwifery education (2021; 2022; 2022), where peer‐supported learning enhanced both learner experience and patient care. However, this finding needs to be balanced against some reported difficulties in directly supervising clinical skills, suggesting that while the model could help address both educational capacity and service delivery challenges simultaneously, careful consideration needs to be given to moment‐by‐moment quality assurance (Smith et al. 2025). This is the point at which physiotherapy evidence diverges from, rather than confirms, the nursing and midwifery literature. In those disciplines, gains in contact volume and continuity carry no comparable (additional) supervisory cost; under the CSP's hands‐on assessment expectations, the same gains directly attenuate the PE's capacity for real‐time skills assessment.

From the participants' point of view, the development of collaborative working relationships and support networks proved crucial to the coaching model's success. Learners' reported increases in confidence in multi‐disciplinary team interactions, which suggests that the approach may better prepare them for contemporary healthcare environments, where interdisciplinary working is increasingly important. This aligns with Vuckovic and Landgren's (2021) findings in psychiatric nursing placements, where they identified enhanced team‐working capabilities as a key outcome of peer‐learning approaches. The strong educational partnership between clinical educators and university staff was also vital, particularly in providing the training and support needed for successful implementation. The findings are also consistent with recent work informed by Communities of Practice theory (Tummons 2025), wherein peripheral participation is seen to be progressively replaced by fuller and more autonomous engagement as learners develop. The PE's role, on this reading, is the reification of a participation trajectory, establishing the conditions, groupings and rotations through which peripheral learners move toward fuller participation, rather than the direct transmission that the one‐to‐one model centred. What the present data add to this framework is the finding that the trajectory is not frictionless: P1's account shows it must be actively managed across ability differentials, and Theme 3 underscores how it can advance learner participation while simultaneously constraining the PE's own oversight. The data above provides a grounded account of the specific conditions (institutional, relational, and logistical) under which these theoretical dynamics are either enabled or constrained within physiotherapy specifically, a context whose regulatory structure makes knowledge transfer from nursing and midwifery evidence something that should be done with considerable care.

Of particular note is how the findings above elaborate upon existing knowledge regarding peer learning dynamics in healthcare education. While Austria et al. (2013) have highlighted the benefits of peer support in nursing education, the findings above reveal more nuanced challenges around managing multiple learners across different physical spaces and coordinating with other healthcare professionals. This suggests that future implementations should consider not only the educational design but also the practical logistics of space and interprofessional working.

### 4.1 Limitations

These findings should be considered within certain limitations. The study focused on PEs who volunteered to participate in the coaching trial, potentially representing those more open to educational innovation. Additionally, while the sample size was appropriate for a qualitative study of this order, experiences may vary in different clinical contexts or with different learner group compositions. This study was set in one geographic area within a single administrative trust and managed by a single HEI. As Williamson and colleagues note in their systematic review of collaborative learning in nursing (2020), contextual factors such as these can significantly influence how lessons from specific peer‐learning initiatives might be applied elsewhere. Transferability of findings to other contexts should be treated with caution, as is always the case with exploratory work. Specifically, the particular HEI‐trust relationship, the level of pre‐implementation support provided to PEs, and the organisational culture of the participating Trust are all likely to have been enabling conditions whose absence elsewhere could substantially alter outcomes.

Moreover, as all participants had chosen to engage in the coaching trial, the sample is likely to reflect PEs who were, at minimum, not actively resistant to the model. The small number who declined participation, or who were not involved in the trial, may hold perspectives that diverge considerably from those captured here; the findings should accordingly be understood as reflecting a context of relative openness to the intervention, rather than the full spectrum of likely PE responses.

Taken together, these limitations suggest that the findings are best understood as providing contextually specific exploratory evidence that C‐PAL is feasible and broadly well‐received under favourable conditions in physiotherapy, rather than as a generalisable account of how PEs will respond to this model across the range of clinical and institutional environments in which physiotherapy placements operate.

### 4.2 Future Research

Further research might productively explore how these findings translate to different clinical settings or learner group configurations. More targeted investigation of the identified logistical challenges could help develop practical solutions for wider implementation. Additionally, exploration of how PE's preparation and support mechanisms could be optimised would be valuable, particularly given the crucial role of educational partnerships identified in this study. As Tulleners et al. (2022) suggest in their work on clinical supervision, the development of robust support structures is essential for the sustainable implementation of innovative supervisory approaches. Finally, one might consider the emergent issues of coaching, peer‐learning and patient safety. While a limited medical literature has suggested that a coaching approach can actively improve quality and safety in some contexts (Mort et al. 2017), the broadly positive findings of the current study warrant further examination in the physiotherapy domain before any firm conclusions can be drawn.

## 5 Implications of Physiotherapy Practice

This study makes a twofold contribution to the existing evidence base. Firstly, it extends C‐PAL research, previously located largely in nursing and midwifery, to an area in which the regulatory framework and historical supervision model are substantively distinct. The CSP's 1000‐h requirement and longstanding one‐to‐one PE model represent a more conservative baseline against which the intervention's feasibility can be assessed. Secondly, unlike the majority of prior studies, this work focuses specifically on the PE's experience rather than the learner's, addressing a recognised gap in how these models are implemented and sustained in practice.

Given the above, the preliminary findings presented here suggest that a coaching model of supervision may represent a feasible alternative under supportive conditions, rather than a demonstrated one; its relevance to wider healthcare education remains a provisional finding for confirmatory work and not a definitive outcome of this study.

Ultimately, however, the approach was reported by participants to have facilitated more extensive patient contact. The participating PEs observed that their charges had gained accelerated clinical reasoning skills, increased autonomy, and improved interdisciplinary collaboration. Notably, the approach was also reported by participants to have facilitated improved continuity of care, suggesting benefits that extend beyond educational objectives to direct service delivery improvements.

The model is not, however, without practical complexity. Logistical challenges, particularly around managing learners across different physical spaces and/or groups of ‘mixed ability’, and coordinating with multidisciplinary teams, require careful consideration in future applications and PE training design. Moreover, the evidence in this paper demonstrates that the success of such an approach pivots critically on robust educational partnerships, particularly between PEs and university staff, and comprehensive preparation of the PEs themselves.

As healthcare systems face increasing pressure to expand training capacity while maintaining educational quality, findings from exploratory studies such as this one contribute to a developing evidence base for alternative supervision models. This single‐site, volunteer‐based study cannot establish that peer‐learning approaches resolve resource constraints, but it offers grounded evidence that they are workable, and valued by educators, where institutional conditions support them.

## Author Contributions

**Sarah Smith:** conceptualization, data curation, funding acquisition, formal analysis, investigation, methodology, project administration, resources, supervision, visualisation, validation, writing – original draft, writing – review and editing. **Adele Anderson:** conceptualization, investigation, methodology, project administration, resources, validation, writing – review and editing. **Simon Godley:** conceptualization, data curation, formal analysis, investigation, methodology, resources, supervision, visualisation, validation, writing – review and editing. **Paul K. Miller:** conceptualization, data curation, funding acquisition, formal analysis, investigation, methodology, project administration, supervision, visualisation, validation, writing – original draft, writing – review and editing.

## Funding

This research was supported by a small grant from the University of Cumbria's Internal Research Fund (IRF) for transcription costs.

## Ethics Statement

Ethical approval for this study was granted by the University of Cumbria Research Ethics Panel (ref: 20/04). All participants provided written consent to participate in advance of their interviews and reaffirmed this consent verbally at the beginning of the recorded interviews. In line with ethical approval, this consent addressed extracts from their qualitative contributions being reported in redacted form in formal published outputs (i.e., journal articles, conference presentations) but not full transcripts being archived for wider consumption (see below).

## Conflicts of Interest

The authors declare no conflicts of interest.

## Supporting information


Supporting Information S1

## Data Availability

Conditions of ethical approval preclude the archiving of the full qualitative data set collected for this study, given the potential for identifiability of participants within a delimited professional community.
