# Supplementary material for: Employing a Coaching Model of Supervision During Physiotherapy Placements: A Qualitative Study of the Practice Educator Experience
Source: Physiother Res Int. 2026 Jun 30;31(3):e70275. doi: 10.1002/pri.70275 (PMC13318423; doi:10.1002/pri.70275)
Supplement: Supplementary file 1 — Supporting Information S1 [file PRI-31-e70275-s001.docx]

**Physio Coaching: Schedule – Educators**

*Encourage the provision of examples from experience, rather than conjecture, throughout.*

| **1** | **Discussion Point** | **Prompts** |
| --- | --- | --- |
| **2** | Can you tell me what you current role is and a little about your responsibilities? |  |
| **3** | Can you tell me what your band is? |  |
| **4** | Have you ever supervised physiotherapy students in the past, can you tell me about that experience? | Roughly how many?  Do you enjoy supervising and teaching? |
| **5** | Roughly how many students would you say you supervise over the year? | Is it your choice to take students or are you allocated? |
| **6** | Had you heard of the term ‘coaching supervision’ before being involved in this project? | Had you considered how you supervise students or does everyone in the dept work the same way with students? |
| **7** | How many students did you support as part of the trial? | 2:1  3:1  4:1 |
| **8** | What thoughts did you have about supervising students using the coaching model? Did you have any pre-conceptions or concerns? | Fear  Anxiety |
| **9** | After completing a coaching placement can you tell me what are your thoughts are about specific moments? | Did it take up a lot more time?  Do you think the patients benefitted? If so, how? |
| **10** | How do you think it compares to the traditional 1:1 supervision? | Did it take up more time?  Was it harder/easier?  Anything that was particularly fun or challenging? |
| **11** | Tell us about the support you received from the University of Cumbria to:  Prepare for the placement?  Work through the placement? | Was there an induction?  Did you feel you were able to reach out if needed?  Was there to much support?  Did the students need the support? |
| **12** | How useful a resource were the learning logs for the students? | Why? |
| **13** | Can you tell me how do you feel the students coped with this style of supervision? | Did it work better than traditional approach?  Any signs of stress or difficulty?  Were you able to spot weakness/strengths in students easier? |
| **14** | Would you use coaching - or any particular parts of it - again? |  |
